# Intestinal microbiome profile of the brown rock sea cucumber (*Holothuria glaberrima*) using ITS and 16S rDNA amplicons from direct mechanical, enzymatic, and chemical metagenomic extraction

**DOI:** 10.1128/mra.00293-25

**Published:** 2025-05-28

**Authors:** Rene Nieves-Morales, Jessica Alejandra Paez-Diaz, Sofia Marie Rodriguez-Carrio, Gabriela Melendez Martinez, Edwin Omar Rivera-Lopez, Josué Rodríguez-Ramos, José E. García-Arrarás, Carlos Rios-Velazquez

**Affiliations:** 1 Microbial Biotechnology and Bioprospecting Laboratory, Biology Department, University of Puerto Rico at Mayagüez, Mayagüez, USA; 2 Department of Food Science, The Pennsylvania State University 311285, University Park, Pennsylvania, USA; 3 Pacific Northwest National Laboratory, Biological Sciences Division, Richland, Washington, USA; 4 Department of Biology, University of Puerto Rico, Río Piedras Campus, Río Piedras, Puerto Rico, USA; Montana State University, Bozeman, Montana, USA

**Keywords:** microbiome, *Holothuria glaberrima*, ITS, 16S, amplicons, direct DNA extraction

## Abstract

Using direct mechanical, enzymatic, and chemical extraction methods, the intestinal microbiome of the marine invertebrate *Holothuria glaberrima* was obtained. ITS and 16S rDNA regions were sequenced to enrich and investigate the prokaryotic and fungal diversity profiles from different anatomical regions within the sea cucumber’s intestinal biology.

## ANNOUNCEMENT

Extraction and sampling methods can introduce biases in DNA analysis, altering taxonomic composition (1). While commercial DNA extraction kits have largely replaced conventional methods, they yield lower amounts of high-quality genetic material (1). Direct extraction methods offer advantages in preserving the genomic profile by minimizing manipulation and getting high-quality and yield DNA (1). Sample size and properties significantly impact DNA quality, highlighting the benefits of direct extraction for better genomic representation (2, 3). Mechanical methods are often associated with higher DNA yields (4), whereas chemical and enzymatic treatments can improve the recovery and integrity of prokaryotic DNA (5).

Most sea cucumber microbiomes rely on commercial kits (6, 7). Therefore, a direct extraction method was employed to unravel microbial communities for further comparative taxonomical and functional studies. *H. glaberrima*, an echinoderm found in Caribbean rocky-intertidal regions (8, 9), regenerates its visceral organs following evisceration, which can be triggered by environmental disturbances (10, 11).

Thirteen specimens were collected from Piñones Beach, San Juan, Puerto Rico (18.451141, −65.905634) on August 5, 2022. They were maintained in a marine water aquarium before inducing evisceration (1, 10, 12). Samples were categorized into complete digestive system (DS), washed intestine (WI), wash contents (CW), and fecal content (FC). Metagenomic DNA was extracted using mechanical (freeze and thaw), enzymatic (lysozyme), and chemical (sodium dodecyl sulfate [SDS] and guanidinium isothiocyanate [GITC] as a chaotropic agent) methods (13–15). Corresponding sets of DNA samples were pooled (9 samples for FC, 6 WI, 6 CW, and 7 DS) in equimolar concentrations for a more representative baseline profile of microbial communities (16–18). The presence and integrity of the pooled DNA were confirmed by gel electrophoresis. For 16S rDNA sequencing, primers 341F (CCTACGGGNGGCWGCAG) and 785R (GACTACHVGGGTATCTAATCC) (19), and ITS1-2 amplification was performed with ITS1F (CTTGGTCATTTAGAGGAAGTAA) and ITS2R (GCTGCGTTCTTCATCGATGC) primers (20, 21). Libraries were normalized to a final concentration of 4.0 nM and sequenced on Illumina MiSeq using 30 cycles.

Data were analyzed using QIIME2 (amplicon-2024.5) (22) with default parameters, except otherwise noted. Cutadapt was used for primer removal (23). DADA2 (1.30) performed denoising (24) with parameters set to “p-trunc-len 141” for 16S rDNA, and “p-trunc-len 113” for ITS. This yielded 659,193 quality reads for 16S rDNA and 478,708 for ITS (Table 1). Data were clustered into 2,296 amplicon sequence variants (ASVs) for 16S rDNA and 2,210 for ITS at a 100% similarity threshold.

**TABLE 1:** Number of reads and quality score per sample provided by QIIME-2

Sample ID	Input	Output	Percentage of input passed the filter	Denoised	Non-chimeric	Percentage of input non-chimeric
DS – ITS	83,682	79,234	94.68	79,107	76,755	91.72
WI – ITS	73,514	69,237	94.18	69,001	68,804	93.59
CW – ITS	134,954	129,273	95.79	128,907	127,238	94.28
FC – ITS	186,558	183,417	98.32	181,753	178,491	95.68
DS – 16S	145,154	139,197	95.90	138,687	135,837	93.58
WI – 16S	129,080	124,816	96.70	124,242	119,179	92.33
CW – 16S	125,795	121,532	96.61	120,548	113,981	90.61
FC – 16S	259,164	248,850	96.02	247,847	239,281	92.33

Taxonomy was assigned using the q2-feature-classifier (25) and classify-sklearn (v.1.4.2) (26) along the UNITE database (unite_ver10_dynamic_04.04.2024-Q2-2024.5.qza) (27) for ITS, and SILVA (classifiers/silva-138-99-nb-classifier.qza) (28) for 16S.

**Fig 1:**
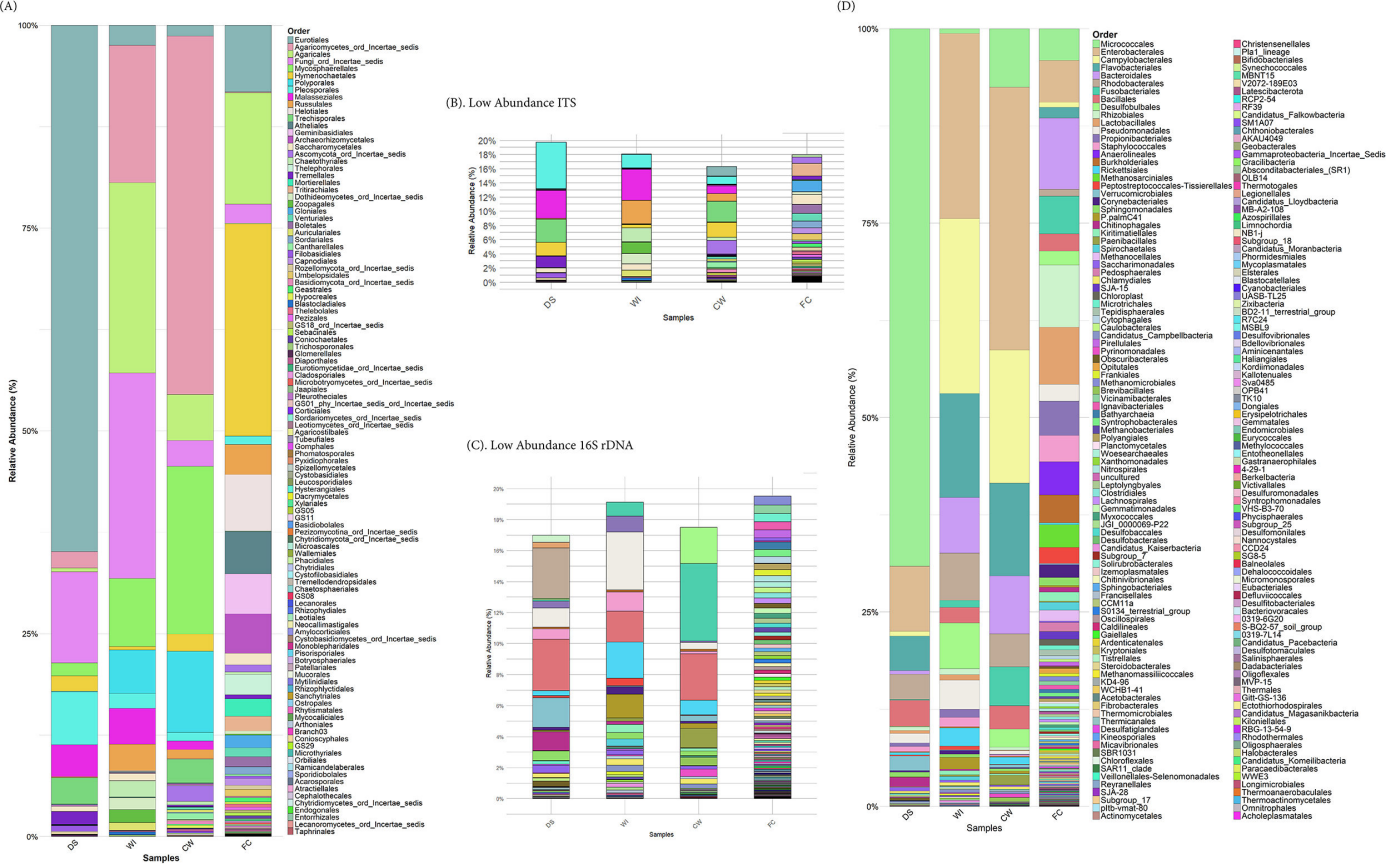
Distribution of fungal and bacterial communities across the complete digestive system (DS), washed intestine (WI), contents from the wash (CW), and fecal content (FC) of *H. glaberrima*. (A–B) Fungal taxa (ITS) bar plots: (**A**) fungal taxa bar plots showing relative abundance at the order level. (**B**) Fungal taxa bar plots highlighting low-abundance order not visible in panel A. (C–D) Bacterial taxa (16S rDNA) bar plots: (**C**) bacterial taxa bar plots displaying low abundance at the order level of panel D. (**D**) Bacterial taxa bar plots showing the relative abundance at the order level.

ITS analysis (Fig. 1A and B) showed that the most abundant fungal orders were as follows: in DS, *Eurotiales* (64.9%) and unclassified fungi (11.2%); in WI, unclassified fungi (25.3%) and *Agaricales* (23.5%); in CW, *Agaricomycetes* (44.2%) and *Mycosphaerellales* (20.7%); and in FC, *Hymenochaetales* (26.3%) and *Agaricales* (13.7%). The 16S analysis (Fig. 1C and D) revealed that the most abundant orders were DS, *Micrococcales* (69.1%) and *Enterobacterales* (8.3%); WI, *Enterobacterales* (23.8%) and *Campylobacterales* (22.5%); CW, *Enterobacterales* (33.8%) and *Campylobacterales* (17.1%); and FC, *Bacteroidales* (9.2%) and *Rhizobiales* (8.0%). This initial taxonomic baseline will allow studies to potentially correlate the roles *H. glaberrima’s* microbiome could have in regeneration.

## Data Availability

The raw sequencing data from this project have been deposited in the National Center for Biotechnology Information (NCBI) under the BioProject accession number: PRJNA1233886. Below are the sample barcodes and SRA accession numbers for the ITS and 16S sequencing data, respectively. ITS sequencing data: *H. glaberrima*, complete digestive system—DS (barcode: ATCGATGC; SRA: SRS22862922) *H. glaberrima*, washed intestine—WI (barcode: ATCGCCTT; SRA: SRS22862921) *H. glaberrima* contents from the washed intestine—CW (barcode: ATCGCCAA; SRA: SRS22862923) *H. glaberrima*, fecal content—FC (barcode: ATCGCGAT; SRA: SRS22862924) 16S sequencing data: *H. glaberrima*, complete digestive system—DS (barcode: CATGCGCA; SRA: SRS22862922) *H. glaberrima*, washed intestine—WI (barcode: CCACGGTG; SRA: SRS22862921) *H. glaberrima* contents from the washed intestine—CW (barcode: CATGGAAG; SRA: SRS22862923) *H. glaberrima*, fecal content—MP1-FC (barcode: CCAGGACT; SRA: SRS22862924) *H. glaberrima*, fecal content—MP2-FC (barcode: CCAGCGGT; SRA: SRS22862924) *H. glaberrima*, fecal content—MP3-FC (barcode: CCAGCTCA; SRA: SRS22862924)
